# Intraspecific variation in the growth and survival of juvenile fish exposed to *Eucalyptus* leachate

**DOI:** 10.1002/ece3.757

**Published:** 2013-09-12

**Authors:** John R Morrongiello, Nicholas R Bond, David A Crook, Bob B M Wong

**Affiliations:** 1 School of Biological Sciences, Monash UniversityMelbourne, Victoria, 3800, Australia; 2 eWater Cooperative Research CentreCanberra, ACT, 2601, Australia; 3 Department of Sustainability and Environment, Arthur Rylah Institute for Environmental Research123 Brown Street, Heidelberg, Victoria, 3084, Australia

**Keywords:** Blackwater, dissolved organic carbon, local adaptation, *Nannoperca australis*, plant secondary metabolite

## Abstract

Whilst changes in freshwater assemblages along gradients of environmental stress have been relatively well studied, we know far less about intraspecific variation to these same stressors. A stressor common in fresh waters worldwide is leachates from terrestrial plants. Leachates alter the physiochemical environment of fresh waters by lowering pH and dissolved oxygen and also releasing toxic compounds such as polyphenols and tannins, all of which can be detrimental to aquatic organisms. We investigated how chronic exposure to *Eucalyptus* leaf leachate affected the growth and survival of juvenile southern pygmy perch (*Nannoperca australis*) collected from three populations with different litter inputs, hydrology and observed leachate concentrations. Chronic exposure to elevated leachate levels negatively impacted growth and survival, but the magnitude of these lethal and sublethal responses was conditional on body size and source population. Bigger fish had increased survival at high leachate levels but overall slower growth rates. Body size also varied among populations and fish from the population exposed to the lowest natural leachate concentrations had the highest average stress tolerance. Significant intraspecific variation in both growth and survival caused by *Eucalyptus* leachate exposure indicates that the magnitude (but not direction) of these stress responses varies across the landscape. This raises the potential for leachate-induced selection to operate at an among-population scale. The importance of body size demonstrates that the timing of leachate exposure during ontogeny is central in determining the magnitude of biological response, with early life stages being most vulnerable. Overall, we demonstrate that *Eucalyptus* leachates are prevalent and potent selective agents that can trigger important sublethal impacts, beyond those associated with more familiar fish kills, and reiterate that dissolved organic carbon is more than just an energy source in aquatic environments.

## Introduction

Environmental gradients such as variation in hydrological or physiochemical conditions can act as important selective agents in freshwater systems (Lake 2003; Lytle and Poff 2004). Many studies have focussed on assemblage-level changes or among-species comparisons along freshwater environmental gradients; generally, species richness declines and resistance and resilience traits become more common in increasingly harsh environments (Schlosser 1990; Poff and Allan 1995; Fritz and Dodds 2005; Crook et al. 2010). Less well appreciated is that spatial and temporal variation in stressors can also lead to divergent local adaptation or significant trait plasticity among populations within a species (Kawecki and Ebert 2004). A growing body of evidence suggests that such intraspecific patterns are likely to be common in fresh waters (Lind et al. 2008; Lytle et al. 2008; Morrongiello et al. 2012).

One such natural stressor prevalent in many freshwater environments worldwide is dissolved organic carbon (DOC) leached from terrestrial vegetation. Whilst this mobilised organic carbon plays a critical role in underpinning many aquatic food webs (Junk et al. 1989; O'Connell et al. 2000; Reid et al. 2008) and more coarse material provides habitat for aquatic organisms (Crook and Robertson 1999), DOC can also be stressful to aquatic organisms via three main pathways.

The first, and most well appreciated, pathway by which organic carbon can act as a stressor is due to rapid microbial consumption of DOC that also rapidly consumes dissolved oxygen in the water (Hladyz et al. 2011; Whitworth et al. 2012). High DOC concentrations can cause “blackwater” events, where waterbodies become hypoxic (dissolved oxygen levels <2 mg L^−1^) and acutely stressful, causing mass mortality of aquatic organisms (Townsend and Edwards 2003; McNeil and Closs 2007; King et al. 2012). Higher temperatures promote microbial activity and greatly increase the probability of hypoxia (Hladyz et al. 2011). The second pathway involves dissolved humic substances in DOC such as humic acid and fulvic acid reducing water pH (Collier and Winterbourn 1987; Gehrke et al. 1993), which in turn can affect growth, survival and reproduction of aquatic biota through impeded ionoregulation (Wood et al. 2003; Hwang and Lee 2007; Barth and Wilson 2010). Humic substances can also interfere with chemical communication and cause hormone-like effects (Fisher et al. 2006; Steinberg et al. 2006). The third stress pathway occurs when toxic organic compounds in DOC such as polyphenols and tannins (plant secondary metabolites) leach into waterways. Exposure to these compounds cannot only be directly lethal to aquatic biota (Rey et al. 2000; McMaster and Bond 2008; Watkins et al. 2011), but also impact on their growth (Canhoto and Laranjeira 2007), development (Martin and Blossey 2013), ability to respire (via gill damage; Temmink et al. 1989; Gehrke et al. 1993) and reproduction (Morrongiello et al. 2011).

Across much of Australia, river red gums (*Eucalyptus camaldulensis*) are a major source of carbon in streams (Francis and Sheldon 2002). Red gum leachate contains over 90 chemical compounds, some of which are toxic to aquatic organisms (Hillis 1966; Cadahía et al. 1997; Conde et al. 1997; Farah et al. 2002). In lowland rivers and ephemeral streams, rain events and flood waters leach carbon compounds from accumulated red gum litter in dry river beds and on floodplains. Subsequent DOC concentrations are largely determined by the frequency of such wetting events, and the extent of riparian vegetation contributing to litter fall (Boulton and Lake 1992; Watkins et al. 2010; Hladyz et al. 2011), with a prominent leachate pulse often occurring in late winter and spring on the first flow events after sufficient leaf biomass has accumulated over summer (Whitworth et al. 2012). These early season flow pulses typically occur when low temperatures limit respirations rates, and thus, whilst hypoxia is generally prevented (Whitworth et al. 2012), organisms can still be exposed to toxic polyphenols.

Importantly, late winter and spring flow pulses can be breeding cues for native fish in south-east Australia (Humphries et al. 1999; Morrongiello et al. 2012), thus creating the potential for their early life-history stages to be exposed to leachate-related stress. Whilst the fitness consequences of acute exposure to lethal *Eucalyptus* leachate concentrations in fish are obvious and documented (Gehrke et al. 1993; McMaster and Bond 2008), significant sublethal impacts in young individuals such as retarded growth, altered physiology or behavioural changes are also likely at lower concentrations and over more chronic exposure periods. These sublethal effects can be proximate or continue to manifest throughout an individual's lifetime and impact on future fitness through, for example, reduced lifespan (Metcalfe and Monaghan 2001), reproductive potential (Taborsky 2006), dispersal ability (Clark et al. 2005) or increased predation risk (Kersten et al. 1991). At present, it is unclear whether populations that are frequently exposed to high *Eucalyptus* leachate concentrations (for example from low/infrequent runoff and high litter fall) exhibit a greater ability to deal with given exposure levels and what the implications of prolonged exposure (weeks to months) may be.

**Figure 1:**
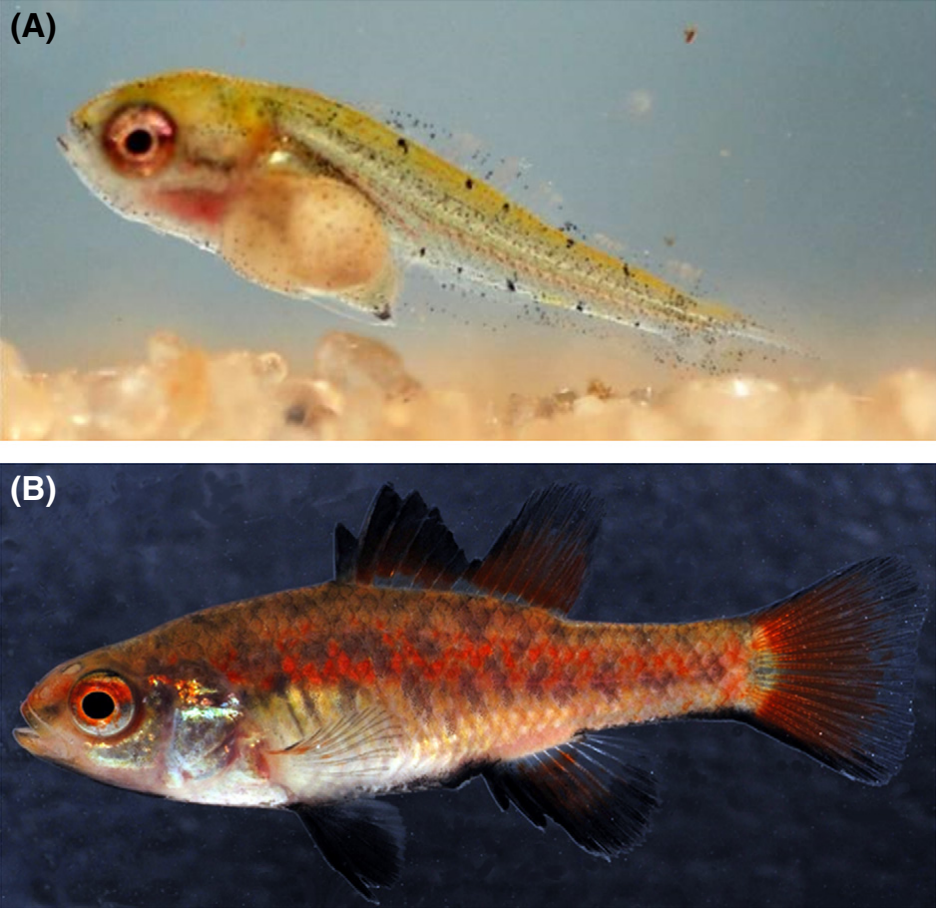
(A) A 2-week-old preflexion southern pygmy perch larva and (B) an adult male southern pygmy perch from Castle Creek, Victoria.

The southern pygmy perch (*Nannoperca australis*, Fig. 1) is a small (<9 cm) freshwater fish found in restricted regions of south-east Australia, inhabiting a range of habitats including perennial streams, large rivers, intermittent creeks and wetlands. Populations of this species are exposed to substantial spatial variation in *Eucalyptus* leachate concentrations (McMaster and Bond 2008) and also differ in the timing of reproduction (late winter to spring: Morrongiello et al. 2012), giving rise to disparity in the risks of leachate exposure during early life stages which are frequently susceptible to chemical stress (e.g. Gehrke et al. 1993; Rasanen et al. 2003). There is also limited gene flow among populations (Cook et al. 2007), increasing the likelihood of adaptive divergence in leachate tolerance in accordance with exposure magnitude.

Here, we expose juvenile southern pygmy perch from populations that experienced different natural exposure rates to reciprocal levels of *Eucalyptus* leachate and asked two questions: (1) what were the lethal and sublethal effects of chronic leachate exposure, independent of hypoxia? and (2) was the magnitude of these stress responses dependent on source population exposure? We hypothesised that exposure to elevated leachate concentrations would not only cause direct mortality, but also impact on growth and that those populations with higher natural leachate exposure would perform better under high leachate concentrations in the laboratory.

## Methods

### Study sites and fish collection

Two hundred and nine juvenile (0.69–1.29 cm) southern pygmy perch (Broken River [*n* = 34], Castle Creek [*n* = 87] and Nine Mile Creek [*n* = 88]) were collected using dipnets from three streams in north-eastern Victoria, Australia, in October 2008. All fish had begun exogenous feeding, but some still possessed the remnants of a yolk sac. The streams differed in a number of hydrological and catchment parameters, with Broken River having a more disturbed riparian zone and substantially, higher and more permanent flow compared with Castle Creek and Nine Mile Creek (Table 1). At the time of collection (10 am–3 pm), all streams were flowing and oxygen levels (>7 mg L^−1^), measured using a Horiba multiparameter probe (Horiba Instruments Inc., Irvine, CA), were significantly higher than that known to cause hypoxia-related stress in southern pygmy perch (<2.3 mg L^−1^ McNeil and Closs 2007; McMaster and Bond 2008).

**Table 1:** Environmental characteristics of three source populations analysed in this study

Site	Latitude	Longitude	MDF (ML)	ZFD	Catchment area (ha)	Riparian vegetation	Polyphenol level
Broken River	36^°^58′33˝S	146^°^6′28˝E	119.8	8.7	21,401	None	0.78 mg L^−1^
Castle Creek	36^°^51′58˝S	145^°^35′9˝E	70.4	23.6	3691	Remnant *Eucalyptus camaldulensis*	22.5 mg L^−1^
Nine Mile Creek	36^°^47′57˝S	145^°^27′15˝E	67.9	35.1	5233	Remnant *Eucalyptus camaldulensis*	4.85 mg L^−1^

MDF, mean daily flow; ZFD, annual number of days with zero flow; Riparian vegetation, qualitative description of trees currently found along riparian zone for approximately 10 km upstream of site; polyphenol level, estimate of polyphenol concentration measured as gallic acid equivalent.

### Leachate measurement and treatment preparation

A 500-mL water sample was taken from each site when fish were collected and returned to the laboratory for analysis of polyphenol concentration (proxy for toxic compounds found in leachate). Samples were immediately chilled and analysed within 2 days of collection. Polyphenol concentration was colorimetrically measured as gallic acid equivalents using a modification of the Folin–Ciocalteau method (Forrest and Bendall 1969). Initially, 0.5 mL of 40 μm filtered stream water was mixed with 0.5 mL of Folin-Ciocalteau reagent (Sigma–Aldrich, Castle Hill, NSW, Australia) and buffered with 5 mL of 1 mol L^−1^ Na_2_CO_3_ in a 25-mL test tube. These tubes were agitated then allowed to react for 1 h. Subsequently, sample absorbance was measured at 765 nm on a Cary 50 UV–Vis spectrophotometer (Varian Inc., Walnut Creek, CA) and compared with gallic acid standards. The point estimate water samples from the three sites differed in polyphenol concentrations (Table 1), and it is likely these differences persisted (if not in exact magnitude) through time due to riparian vegetation and hydrological differences among sites (Table 1) and because temporally replicated field-based measures of light transmission through the water column were consistent in these sites (Morrongiello et al. 2010).

Based on these field measurements, juveniles were exposed to three initial leachate concentrations (low: 1 mg L^−1^ polyphenol; medium: 5 mg L^−1^ polyphenol; and high: 20 mg L^−1^ polyphenol). The lowest concentration represented an ecologically relevant, baseline control as no stream in this region would have zero levels of leachate (pers. obs.). Dried river red gum leaves collected from the Castle Creek floodplain had previously been submerged in black tubs for 20 days to ensure maximum leachate concentration (O'Connell et al. 2000). This concentrate was filtered, its polyphenol concentration calculated as above, and then diluted down to the three treatment levels using filtered aquarium water.

### Experimental procedure

Fish from each population (Broken low: *n* = 10; medium: *n* = 13; high: *n* = 11; Castle low: *n* = 29; medium: *n* = 28; high: *n* = 30; Nine Mile low: *n* = 28; medium: *n* = 30; high: *n* = 30) were exposed to each of the three treatment levels in a fully crossed design to explore potential differences in local population responses to chronic leachate exposure. Individual fish were placed in 500-mL jars in a temperature control room (16°C ± 1°C, 12:12 h light–dark photoperiod) containing gravel substrate and allowed to acclimate for 2 days. This temperature is cooler than those often associated with leachate-induced hypoxia (≥20°C) in temperate Australian streams (Hladyz et al. 2011; Whitworth et al. 2012). Leachate treatments were applied on day 3. The toxicity of leachate, and in particular polyphenols, is reduced through time due to oxidative polymerisation and photo- and bio-degradation (Temmink et al. 1989; Serrano 1994; Howitt et al. 2008). The actual leachate levels experienced by fish in this experiment would therefore naturally decline through time so we reapplied treatments and undertook 100% water changes on days 30, 71 and 94 of the experiment. This treatment regime aimed to simulate a temporally varying leachate exposure history as observed in natural environments (i.e. sharp pulses associated with flow events and gradual declines associated with photo- and bio-degradation; Howitt et al. 2008; Whitworth et al. 2012). Treatment levels, therefore, refer to a maximum, but time-varying, leachate exposure rather than an unnatural constant concentration. Dissolved oxygen was measured on an *ad hoc* basis throughout the study using a Hach luminescent dissolved oxygen probe (Hach Company, Loveland, CO) and remained above obviously stressful levels (>5 mg L^−1^; McNeil and Closs 2007).

Fish were initially fed 5 mL day^−1^ of cultured brine shrimp nauplii (*Artemia* spp.) then after 40 days, 5 mL day^−1^ of concentrated zooplankton (*Daphnia* spp., ostracod spp. and copepod spp.) collected from a nearby lake. Four 5 mL replicates of each food type were randomly sampled on different days, and the number of nauplii and plankton counted. The solution contained 581 ± 40 individuals 5 mL^−1^ (mean ± SE). These prey densities were within those encountered in the natural environment (King 2004). All fish ate these prey items.

The experiment was conducted over 124 days. Fish were monitored daily, and those that had lost equilibrium (could no longer maintain an upright position) were removed from the experiment and considered to have “died”. These fish were then euthanised by an overdose of clove oil. Lost equilibrium is often used as a sublethal endpoint for fish toxicity tests (McNeil and Closs 2007). Fish were photographed on days 1, 10, 18, 29, 49 and 71 and their length (mm) calculated using Image J 1.38 (NIH, http://rsweb.nih.gov/ij/) image analysis software. Specific growth rates (mm mm^−1^ day^−1^) of each individual were calculated as 100× (ln *L*_f_ – ln *L*_i_)/*t* where *L*_i_ and *L*_f_ refer to the initial and final length and *t* is the time interval between consecutive measurements in days.

### Statistical analyses

#### Survival

Kaplan–Meier curves and median product-limit estimators of survival for each population and treatment were used to examine survivorship as a function of time (Tableman and Kim 2005). The relationship between fish *survival* (number of days alive), and the covariates *treatment*, *population* and initial body *length* (length measured at day 1) was analysed using Cox proportional-hazards regression. This semiparametric model deals effectively with time-to-event data characterised by right censoring (death not recorded at trial end) and an unknown, complex or temporally changing underlying distribution (Therneau and Granmbsch 2000). The effect of each covariate multiplies the hazard (instantaneous risk of death) by a constant factor (coefficient), with change in hazard being inversely proportional to likelihood of survival. Importantly, whilst covariate values can change through time, the effect of their coefficients must remain constant (“proportional hazards”). The proportional-hazards model can be extended to allow nonlinear relationships between a time-to-event response and covariates to ensure this assumption is met. One such extension is the fitting of smoothing splines (Therneau and Granmbsch 2000). Interaction terms cannot be fitted between categorical variables and splines, and so interactions were restricted to among *population*, *treatment* and linear *length*. Model assumptions were checked using residual plots and Grambsch and Therneau's test for proportional hazards (Grambsch and Therneau 1994). The model with the lowest AIC corrected for small sample size (AICc) (Burnham and Anderson 2002) was selected as best among candidate models. Analyses were performed using the Survival package (2.36–14) in R 2.15.0 (R Development Core Team 2012).

#### Growth

Predictor variables for an individual's *growth rate* included *population*, *treatment*, *status* (coded “dead” or “alive” at t_n_ depending on survivorship to t_n+1_, which accounted for differential mortality of fish), *length* (*L*_i_ for each temporal estimate, from specific growth formula) and sampling *time* (ordered categorical). Growth rate was analysed using a mixed-effects linear model with a series of random effects structures to account for the data's hierarchical structure, heterogeneity and temporal correlation (Zuur et al. 2009). Firstly, a random intercept (*fish ID*) was included as growth rate estimates were more likely to be similar within, rather than among, individuals. Secondly, we explored patterns of data heterogeneity across predictor variable strata (*population*, *treatment*, *time* and their interactions) using different variance structures. Thirdly, it is likely that growth estimates within an individual are correlated through time, so we fitted a range of temporal correlation structures. Combinations of the above random effects structures were fitted to a fully interactive fixed effects model using restricted maximum likelihood estimation (REML) and the best one selected using likelihood ratio tests. The best random effects structure included a random intercept (*fish ID*), and different variances per *time* stratum indicating that growth was more variable earlier in the experiment (multiplicative factors for standard deviations: day 10: 1; day 18: 0.96; day 29: 0.72; day 49: 0.51; day 71: 0.54) and an autoregressive AR(1) correlation structure for temporal replicates within an individual (phi = −0.324). This random effects structure was adopted in the subsequent exploration of the optimal fixed effects structure.

We implemented a top–down strategy to select the most parsimonious fixed effect structure (Zuur et al. 2009). The adequacy of candidate models fitted with maximum likelihood estimates of error (ML) was compared using AICc. The initial full model included the four-way *population* × *treatment* × *time* × *length* and three way *treatment* × *status* × *length* interactions. The full five-way interaction could not be fitted as no individuals measured on day 71 died (right censored) and only one Broken River individual died. Adherence to model assumptions was checked with the full model and *growth rate* subsequently square-root + 1 transformed to ensure homogeneity of errors. The optimal model was re-analysed using REML to produce unbiased parameter estimates (Zuur et al. 2009). Differences among *population*, *treatment* and *status* levels were assessed using Helmert contrasts and trends across temporal replicates (*time*) were explored using polynomial contrasts (Quinn and Keough 2002; Logan 2010). Analyses were performed using the nlme package (3.1–105) in R 2.15.0.

## Results

### Initial fish size

Broken River juveniles were significantly larger than those from Castle and Nine Mile Creek at time of collection (means [cm]: Broken 1.06 > Castle 0.99 = Nine Mile 0.99, ANOVA *F*_2,206_ = 3.97, *P* = 0.020). There was, however, no difference in fish sizes among the three *treatments* (*F*_2,204_ = 0.28, *P* = 0.76) nor among treatments within populations (*population* × *treatment* interaction *P* = 0.29).

### Survival

**Figure 2:**
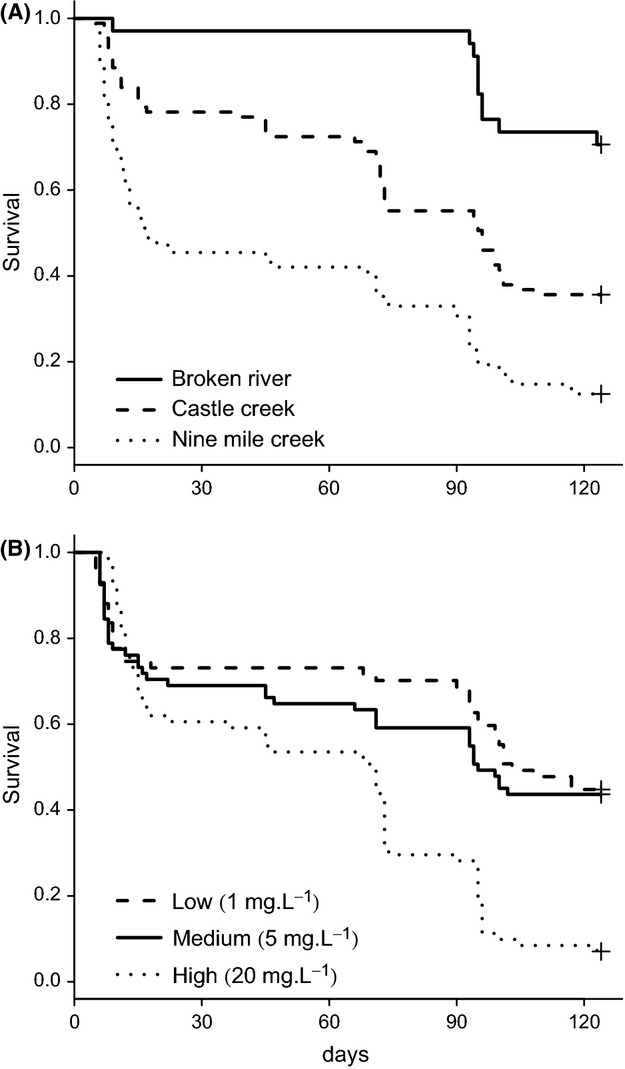
Kaplan–Meier survival curves showing estimated survivorship through time of juvenile southern pygmy perch for: (A) three source populations and (B) three *Eucalyptus* leachate treatment levels. (+) indicates right-censored data.

Kaplan–Meier survivorship curves, pooled across *treatment* and *population* levels, respectively, showed marked variation in survival among populations and treatments with large declines in the early and latter stages of the experiment (Fig. 2A and B). The median survival of Broken River fish was 124 days (71% survival at day 124), Castle Creek fish 96 days (36% at day 124) and Nine Mile Creek 17 days (13% at day 124), whilst low treatment fish had the highest median survival (103 days, 45% at day 124), followed by medium (95 days, 44% a day 124) and then high (71 days, 7% at day 124).

Nineteen models of increasing complexity (including either linear or nonlinear [spline] *length* terms) were fitted to the full data set to predict fish *survival*. The most parsimonious model included *population*, *treatment* and *length* spline. Compared with Broken River, Castle Creek fish were 3.75 times and Nine Mile Creek fish 9.65 times more likely to die during the experiment. There was no difference in the survivorship of low and medium treatments, but high-treatment fish were 3.2 times more likely to die. Bigger individuals also had higher survivorship, but this relationship was nonlinear. Importantly, however, the assumption of proportional hazards was violated for the high treatment in all models and indicated an increasing hazard for these fish through time (Fig. 2B). This increased hazard was associated with a disproportionate mortality response of high-treatment fish to the reapplication of leachate treatments on days 71 and 94. Therefore, we conducted separate analyses on the low and medium data (138 individuals) and the high-treatment data (71 individuals) to more thoroughly explore the effects of source *population* and *length*.

**Figure 3:**
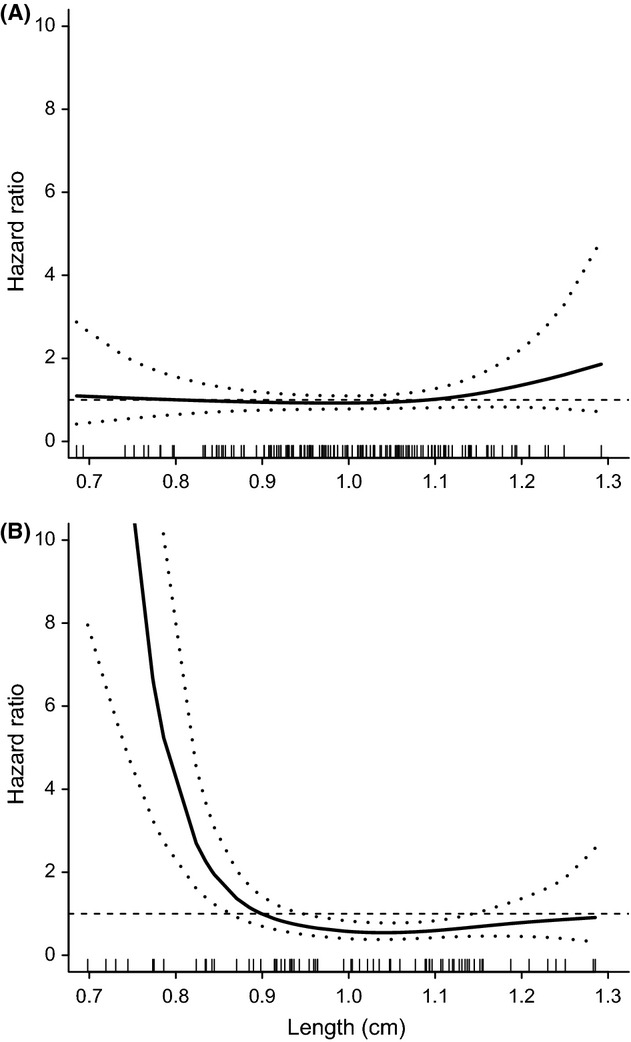
Hazard ratio (±95% confidence interval) as a function of body length for juvenile southern pygmy perch exposed to (A) low and medium and (B) high *Eucalyptus* leachate treatments. The horizontal dashed line is the baseline hazard ratio (=1) and represents no change in survival likelihood due to treatment exposure; higher ratios indicate increased mortality risk and lower ratios decreased mortality risk. “Rug plot” (short vertical lines on *x* axis) illustrates the distribution of observed length values.

The best model for the pooled low and medium treatment survival data included the terms *population* and the nonlinear *length* spline (Table 2). On average, Castle Creek fish were 4.5 times and Nine Mile Creek fish 12.9 times more likely to die than Broken River fish, and there was little evidence of a body size effecting mortality risk (Fig. 3A). Survivorship in the high treatment was also dependent on both source *population* and body *length* (Table 2). *Population* had a similar effect as that observed in the low and high analysis, with both Castle and Nine Mile Creek fish having higher mortality risks when compared with Broken River fish. There was also a strong, nonlinear effect of body size at high leachate exposure (Fig. 3B). Smaller fish had much higher mortality risks with their hazard ratio increasing almost linearly with each unit of length below ∼0.9 cm. Fish bigger than this size had relatively stable mortality risks slightly below 1 (baseline hazard ratio).

**Table 2:** Juvenile southern pygmy perch survival estimated hazard coefficients (multiplicative change in risk) for population and length from Cox regression models (delineated by treatment low: 1 mg L^−1^; medium: 5 mg L^−1^; high: 20 mg L^−1^ polyphenol). Castle and Nine Mile coefficients are hazards relative to Broken River survival. Smoothing spline degrees of freedom (df) estimated from automated selection (1 df = straight line)

			95% CI	
			
Variable	Coefficient (SE)	Wald	Lower	Upper
Low and medium treatments				
Castle	4.536 (1.844)	6.11*	1.39	15.03
Nine Mile	12.936 (1.694)	18.27***	4.06	16.50
Length (spline 1.9 df)		1.57^a^		
High treatment				
Castle	3.525 (1.618)	6.83**	1.38	9.03
Nine Mile	9.679 (1.655)	20.36***	3.60	26.05
Length (spline 2.24 df)		19.38***		

SE, standard error; CI, confidence interval.

* *P* < 0.05,

** *P* < 0.01,

*** *P* < 0.001,

a *P* < 0.2

### Growth

**Figure 4:**
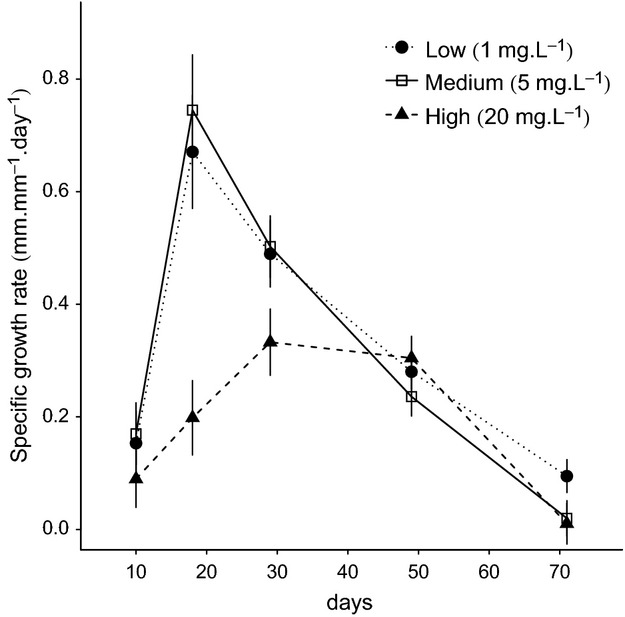
Temporal variation in specific growth rates of juvenile southern pygmy perch exposed to three *Eucalyptus* leachate treatments. Points represent means ± 1 SE.

**Figure 5:**
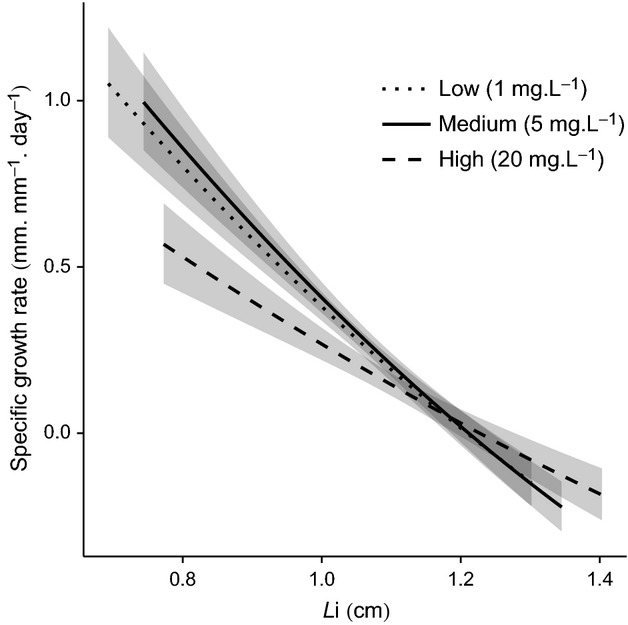
Relationships between body length (*L*_i_ from specific growth rate formula) and predicted specific growth rate (±95% confidence interval) in southern pygmy perch when exposed to three *Eucalyptus* leachate treatments (all other covariates held at mean values).

**Figure 6:**
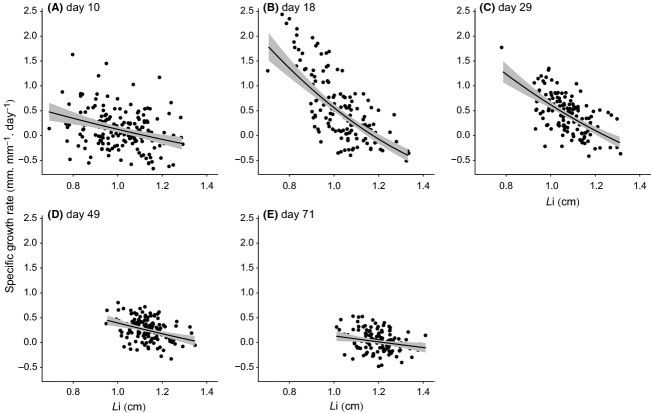
Temporal variation in the predicted relationship between body length (*L*_i_ from specific growth rate formula) and specific growth rate (±95% confidence interval) in southern pygmy perch when exposed to three *Eucalyptus* leachate treatments (all other covariates held at mean values). (A) day 10, (B) day 18, (C) day 29, (D) day 49 and (E) day 71. Points represent observed growth rates at each sample time.

The optimal model explaining variation in fish *growth rate* included *population* (*F*_2,162_ = 13.995, *P* < 0.001), *status* (*F*_1,518_ = 13.128, *P* < 0.001) and the interactions *treatment* × *time* (*F*_8,518_ = 2.307, *P* = 0.019), *treatment* × *length* (*F*_2,518_ = 6.668, *P* = 0.001), and *time* × *length* (*F*_4,518_ = 20.263, *P* < 0.001). Broken River fish on average grew faster than those from Castle Creek which in turn grew faster than those from Nine Mile Creek (0.294 ± 0.020, 0.193 ± 0.013 and 0.183 ± 0.016 mm mm^−1^ day^−1^ SE, respectively; Table 3). For a given time, fish that did not survive until the next measurement had slower growth rates than those that did (0.224 ± 0.023 SE vs. 0.032 ± 0.096 SE; Table 3). Both low- and medium-treatment fish displayed a similar temporal growth trend (Table 3) with a peak in rate at 18 days, followed by a steady growth rate decline to 71 days (Fig. 4). High-treatment fish, however, displayed a different temporal trend to the other treatments (Table 3). There was no growth rate peak at 18 days and slower growth over the first 29 days of the experiment, followed by a growth rate decline to 71 days (Fig. 4). There was a similar negative relationship between length and growth rate for low- and medium-treatment fish, but the slope of this relationship was shallower and the intercept lower for the high treatment (Table 3, Fig. 5). These differences were primarily driven by a retardation of growth among smaller fish in the high treatment. The length by growth rate relationship also varied through time (Table 3; Fig. 6A–E): it initially had a shallow slope and low intercept at day 10, steepened at day 18 and 29, and then shallowed again as fish got older and larger at days 49 and 71.

**Table 3:** Parameter estimates including polynomial trends from optimal mixed-effects model describing variation in juvenile southern pygmy-perch-specific growth rates (sqrt + 1 transformed, mm mm^−1^ day^−1^) as a function of source population, treatment level (low: 1 mg L^−1^; medium: 5 mg L^−1^; high: 20 mg L^−1^ polyphenol), observation time and body length

Parameter	Estimate (SE)	df	*t*-value
Intercept	1.885 (0.049)	518	38.253***
Population			
Castle versus Broken	−0.022 (0.005)	162	4.752***
Nine Mile versus Broken and Castle	−0.009 (0.003)	162	3.408**
Status			
Dead versus Alive	−0.045 (0.012)	518	3.623**
Treatment			
Medium versus low	0.027 (0.057)	162	0.479^b^
High versus low and medium	−0.120 (0.031)	162	3.860**
Time			
Time	−0.621 (0.116)	518	5.350***
Time^2^	−0.535 (0.120)	518	4.443***
Time^3^	0.717 (0.129)	518	5.570***
Length	−0.755 (0.044)	518	17.147***
Treatment × Time (compared with low)			
Medium × Time#	−0.024 (0.014)	518	1.668^a^
High × Time	−0.002 (0.008)	518	0.277^b^
High × Time^2^	−0.002 (0.007)	518	0.340^b^
High × Time^3^	−0.028 (0.008)	518	3.407**
Treatment × Length (compared with low)			
Medium × Length	−0.022 (0.053)	518	0.421^b^
High × Length	0.102 (0.028)	518	3.609**
Time × Length			
Time × Length	0.563 (0.102)	518	5.531***
Time^2^ × Length	0.375 (0.108)	518	3.480***
Time^3^ × Length	−0.620 (0.118)	518	5.266***

# Quadratic and cubic trends not significant thus omitted.

** *P* < 0.01,

*** *P* < 0.001,

a *P* < 0.1,

b *P* < 0.9.

## Discussion

Chronic exposure to elevated *Eucalyptus* leachate levels caused significant lethal and sublethal impacts in juvenile southern pygmy perch, independent of hypoxia. Body size played an important role in determining the strength and direction of these treatment effects. Interestingly, the magnitude of leachate-induced lethal and sublethal effects was also strongly dependent on source population. This suggests that fitness consequences can vary spatially or are dependent on developmental stage. Taken together, these results emphasise the importance of considering both individual and population-level variation across multiple response variables when investigating ecological and evolutionary implications of stressors at the landscape scale.

Long-term patterns in fish growth and survivorship were inter-related and displayed a strong negative dose-dependent response to chronic leachate exposure. Whilst fish in all three treatments experienced mortality, those in the high leachate treatment had a much higher hazard ratio. These fish also displayed an increased mortality risk through time and an increased vulnerability to subsequent leachate pulses, indicating that the cumulative effect of leachate exposure is greater at high doses and that past exposure does not necessarily convey resistance. Such a result implies that leachates can be especially potent stressors in ephemeral systems where larvae are repeatedly exposed to pulses of leachate on sequential flow events (Whitworth et al. 2012), as leachate toxicity is dependent not only on dose but also exposure frequency.

Our novel long-term findings provide a broader ecological context for studies that have focussed on the impacts of short-term, acute leachate exposure on fish. For example, critical dose thresholds in the toxic resistance to DOC and polyphenol exposure were identified for adults of three species of fish (including southern pygmy perch) over a 96-h period (McMaster and Bond 2008). These threshold doses, however, were significantly reduced when stressor exposure was combined with low dissolved oxygen. Likewise, Gehrke et al. (1993) found that 8–15-day-old golden perch (*Macquaria ambigua*) larvae had higher mortality rates after 72 h when exposed to elevated levels of red gum leaf leachate, and this effect again interacted with reduced dissolved oxygen. Both these studies implicated the ability of polyphenols to damage gills (Temmink et al. 1989; Gehrke et al. 1993), and thus interfere with respiration, in explaining mortality patterns. It is highly probable that a similar effect caused much of the mortality observed in our study although the longer time frames in our study suggest that compounds within leachate or associated physiochemical changes can have both acute and chronic effects on fish survival.

Elevated DOC and leachate concentrations in fresh waters can result in lower pH and dissolved oxygen levels, including hypoxia (Howitt et al. 2007). These in turn have been shown to acutely impact on many aquatic organisms, including fish as well as cause larger-scale changes in assemblage structure (Collier and Winterbourn 1987; King et al. 2012). Any reductions in dissolved oxygen and pH associated with the higher leachate treatments in our study also likely played a role in increasing the mortality risk and retarding growth of fish by causing physiological stress (Temmink et al. 1989; Gehrke et al. 1993; Barth and Wilson 2010). It is important to note that we did not manipulate oxygen concentrations, which stayed above hypoxic levels. Thus, additional mortality may have been caused by other compounds or physiochemical changes that may be toxic only after chronic exposure.

High-treatment fish had much slower growth rates than the other two treatments. This was most pronounced early in the experiment and likely has a number of immediate fitness implications for the individual. These include impeded ontogenetic development resulting in reduced swimming performance and thus dispersal potential (Clark et al. 2005), inability to access gape-size limited food (Post 2003) and longer exposure to size-dependent predation (McCormick and Hoey 2004). In the longer term, among-individual growth variation may continue to be manifested through to adulthood and reduce reproductive fitness due to smaller body sizes (Morrongiello et al. 2012) and reduced lifespan due to costs associated with compensatory growth (Inness and Metcalfe 2008).

Body size was an important predictor of fish growth and survivorship. Smaller juveniles (<0.9 cm) were less likely to survive in the high leachate treatment, and growth rate (i.e. increase in fish length over time) was influenced by both leachate exposure and allometric growth (Weatherley and Gill 1987). Fish in the high leachate treatment experienced lower growth rates, and this effect was most pronounced in smaller individuals. A similar result was observed by Gehrke et al. (1993) whereby older and larger golden perch larvae were more resistant to leachate exposure than younger and smaller larvae. Morrongiello et al. (2011) found that leachate exposure limited the ability of southern pygmy perch to reproduce, whilst King et al. (2012) implicated a blackwater event in causing recruitment failure in small-bodied native Australian fish. Taken together, these results indicate that vulnerability to the impacts of *Eucalyptus* leachate is highest in the early life stages of fish.

The magnitude of difference in polyphenol concentration was much larger between medium and high treatments (5 mg L^−1^ vs. 20 mg L^−1^) than between medium and low treatments (5 mg L^−1^ vs. 1 mg L^−1^). Accordingly, there was no detectable difference in growth and survival of fish exposed to the lower two treatments, but the high-treatment fish were adversely affected. There was similar evidence of a nonlinear effect of leachate exposure on the survival of golden perch larvae (Gehrke et al. 1993) and, albeit at extreme levels, in adult mountain galaxias (*Galaxias olidus*), carp gudgeon (*Hypseleotris klunzingeri*) and southern pygmy perch (McMaster and Bond 2008). Our results indicate a tolerance threshold to the effects of chronic leachate exposure in juvenile pygmy perch somewhere between 5 and 20 mg L^−1^ under normoxia. Further experimentation is required to fully parameterise this response curve.

Spatial variation in stressors can result in a differential selection environment and local divergence in fitness-related traits. The likelihood of this local adaptation increases when there is limited gene flow among populations, as occurs in southern pygmy perch (Cook et al. 2007). In our study, however, among-population differences in leachate resistance were not concordant with patterns predicted by local adaptation theory. If leachate resistance was locally adaptive, then each population should perform best under its own local conditions (Kawecki and Ebert 2004), and thus, there should be a significant *population* × *treatment* interaction in each analysis. This was not the case and instead fish from the Broken River, the lowest leachate site, had higher survival and growth than either Castle or Nine Mile Creek fish. It is likely that some populations with more ephemeral flows or increased *Eucalyptus* litter loading will be naturally exposed to even higher leachate concentrations than those observed in Castle Creek for discrete or prolonged time periods. The tolerance thresholds and fitness consequences of leachate exposure in larvae from these populations are unknown, although given our results (lack of detectable local adaptation), they are likely to be similar to those observed here.

There are a number of plausible explanations for spatial variation in leachate resistance. First, and potentially most likely, initial differences in juvenile body size (with Broken River juveniles larger than those from Castle and Nine Mile Creek) could have confounded population effects if Broken River juveniles were also older or more developed, and thus had greater resistance to leachate. Secondly, leachate concentrations will naturally fluctuate through time (sensu Whitworth et al. 2012) such that the point observations made in this study may not be representative of those actually experienced by fish over the longer term. Similarly, current leachate levels may not reflect the historical level of stress to which fish become locally adapted, with for example, the extensive riparian clearing in the Broken River catchment upstream of, and including, the study area resulting in reduced litter loads and thus potentially lower DOC levels (Watkins et al. 2010). Temporal variation in selective regimes tends to favour generalist rather than specialist genotypes (Kawecki and Ebert 2004). Thirdly, the fish were wild caught, and this raises the potential for genetic, maternal or early life experience effects, not accounted for in this study, to influence results. For example, Broken River mothers may be less stressed due to low leachate exposure, allowing them to allocate more resources to each offspring and thus a survival benefit in the transition from endogenous to exogenous feeding (e.g. McCormick 2003). However, this hypothesis would predict larger offspring being produced under low leachate concentrations, yet Morrongiello et al. (2011) found that *Eucalyptus* leachate exposure only affects the probability of southern pygmy perch mothers spawning and not egg size or number. Finally, It could be that our experimental design did not adequately assess the impacts of multiple stressors (Darling and Cote 2008) that can co-occur in the environment with elevated leachate concentrations, such as hypoxia (Gehrke et al. 1993; McMaster and Bond 2008) or lowered pH (Barth and Wilson 2010). However, the reproductive timing of pygmy perch means that larvae and juveniles are likely to be exposed to elevated leachate concentrations independent of hypoxic (Whitworth et al. 2012) or acidic conditions (Watkins et al. 2010), and thus, the single stressor experiment undertaken here is ecologically valid.

In summary, terrestrial plant leachates are a prevalent natural chemical in many freshwater environments, and our results demonstrate how exposure to associated toxic compounds and physiochemical changes to water quality can have significant fitness consequences in aquatic organisms. Interestingly, *Eucalyptus* leachate impacts occurred independently of hypoxia indicating that leachate compounds in DOC can have direct toxic effects on fish. The impact of leachate exposure on juvenile fish was also dependent on source population and the magnitude and timing of exposure, with smaller (and likely younger) fish being more vulnerable. Notwithstanding the limitations of scaling up from laboratory experiments to natural systems, these results provide biological information relevant to applied decisions about stream management (e.g. how and when environmental flows are delivered) in regulated systems with high litter loadings (e.g. Howitt et al. 2007; Hladyz et al. 2011). This is especially pertinent given that spawning by many native fishes can be associated with these same flow events (Humphries et al. 1999). Further work is needed to understand the spatial and temporal dynamics of DOC across the landscape as this will enhance our ability to attribute and predict among-population responses to that variation. Similarly, additional experiments exploring the potential interactive effects of *Eucalyptus* leachate and other physiochemical parameters like dissolved oxygen and pH, as well as a more detailed exploration of specific toxic leachate compounds (e.g. tannins), will help isolate the key drivers of lethal and sublethal responses to DOC. Finally, our results highlight the complexity of ecological interactions occurring on the interface between terrestrial and aquatic environments and the need to consider DOC as more than just an energy source in fresh waters.

## References

[b1] Barth BJ, Wilson RS (2010). Life in acid: interactive effects of pH and natural organic acids on growth, development and locomotor performance of larval striped marsh frogs (*Limnodynastes peronii*. J. Exp. Biol.

[b2] Boulton AJ, Lake PS (1992). Benthic organic matter and detritivorous macroinvertebrates in 2 intermittent streams in south-eastern Australia. Hydrobiologia.

[b3] Burnham KP, Anderson DR (2002). Model selection and inference: a practical information-theoretic approach.

[b4] Cadahía E, Conde E, García-Vallejo MC, Fernandez de Simon B (1997). High pressure liquid chromatographic analysis of polyphenols in leaves of *Eucalyptus camaldulensis*
*E. globulus* and *E. rudis*: proanthocyanidins, ellagitannins and flavonol glycosides. Phytochem. Anal.

[b5] Canhoto C, Laranjeira C (2007). Leachates of *Eucalyptus globulus* in intermittent streams affect water parameters and invertebrates. Int. Rev. Hydrobiol.

[b6] Clark DL, Leis JM, Hay AC, Trnski T (2005). Swimming ontogeny of larvae of four temperate marine fishes. Mar. Ecol. Prog. Ser.

[b7] Collier KJ, Winterbourn MJ (1987). Faunal and chemical dynamics of some acid and alkaline New Zealand streams. Freshw. Biol.

[b8] Conde E, Cadahia E, GarciaVallejo MC (1997). Low molecular weight polyphenols in leaves of *Eucalyptus camaldulensis*
*E. globulus* and *E. rudis*. Phytochem. Anal.

[b9] Cook BD, Bunn SE, Hughes JM (2007). Molecular genetic and stable isotope signatures reveal complementary patterns of population connectivity in the regionally vulnerable southern pygmy perch (*Nannoperca australis*. Biol. Conserv.

[b10] Crook DA, Robertson AI (1999). Relationships between riverine fish and woody debris: implications for lowland rivers. Mar. Freshw. Res.

[b11] Crook DA, Reich P, Bond NR, McMaster D, Koehn JD, Lake PS (2010). Using biological information to support proactive strategies for managing freshwater fish during drought. Mar. Freshw. Res.

[b12] Darling ES, Cote IM (2008). Quantifying the evidence for ecological synergies. Ecol. Lett.

[b13] Farah A, Fechtal M, Chaouch A, Zrira S (2002). The essential oils of *Eucalyptus camaldulensis* and its natural hybrid (clone 583) from Morocco. Flavour. Fragr. J.

[b14] Fisher HS, Wong BBM, Rosenthal GG (2006). Alteration of the chemical environment disrupts communication in a freshwater fish. Proc. Biol. Sci.

[b15] Forrest GI, Bendall DS (1969). Distribution of polyphenols in the tea plant (*Camellia sinesis* L.). Biochem. J.

[b16] Francis C, Sheldon F (2002). River Red Gum (*Eucalyptus camaldulensis* Dehnh.) organic matter as a carbon source in the lower Darling River, Australia. Hydrobiologia.

[b17] Fritz KM, Dodds WK (2005). Harshness: characterisation of intermittent stream habitat over space and time. Mar. Freshw. Res.

[b18] Gehrke PC, Revell MB, Philbey AW (1993). Effects of river red gum, *Eucalyptus camaldulensis*, litter on golden perch, *Macquaria ambigua*. J. Fish Biol.

[b19] Grambsch P, Therneau TM (1994). Proportional hazards tests and diagnostics based on weighted residuals. Biometrika.

[b20] Hillis WE (1966). Variation in polyphenol composition within species of *Eucalyptus* L'Herit. Phytochemistry.

[b21] Hladyz S, Watkins SC, Whitworth KL, Baldwin DS (2011). Flows and hypoxic blackwater events in managed ephemeral river channels. J. Hydrol.

[b22] Howitt JA, Baldwin DS, Rees GN, Williams JL (2007). Modelling blackwater: predicting water quality during flooding of lowland river forests. Ecol. Model.

[b23] Howitt JA, Baldwin DS, Rees GN, Hart BT (2008). Photodegradation, interaction with iron oxides and bioavailability of dissolved organic matter from forested floodplain sources. Mar. Freshw. Res.

[b24] Humphries P, King AJ, Koehn JD (1999). Fish, flows and flood plains: links between freshwater fishes and their environment in the Murray-Darling River system, Australia. Environ. Biol. Fishes.

[b25] Hwang PP, Lee TH (2007). New insights into fish ion regulation and mitochondrion-rich cells. Comp. Biochem. Physiol., Part A Mol. Integr. Physiol.

[b26] Inness CLW, Metcalfe NB (2008). The impact of dietary restriction, intermittent feeding and compensatory growth on reproductive investment and lifespan in a short-lived fish. Proc. Biol. Sci.

[b27] Junk WL, Bayley PB, Sparks RE, Dodge DP (1989). The flood pulse concept in river-floodplain systems. Proceedings of the international large river symposium.

[b28] Kawecki TJ, Ebert D (2004). Conceptual issues in local adaptation. Ecol. Lett.

[b29] Kersten M, Britton RH, Dugan PJ, Hafner H (1991). Flock feeding and food intake in little egrets: the effects of prey distribution and behaviour. J. Anim. Ecol.

[b30] King AJ (2004). Density and distribution of potential prey for larval fish in the main channel of a floodplain river: Pelagic versus epibenthic meiofauna. River Res. Appl.

[b31] King AJ, Tonkin Z, Lieshcke J (2012). Short-term effects of a prolonged blackwater event on aquatic fauna in the Murray River, Australia: considerations for future events. Mar. Freshw. Res.

[b32] Lake PS (2003). Ecological effects of perturbation by drought in flowing waters. Freshw. Biol.

[b33] Lind MI, Persbo F, Johansson F (2008). Pool desiccation and developmental thresholds in the common frog, *Rana temporaria*. Proc. Biol. Sci.

[b34] Logan M (2010). Biostatistical design and analysis using R: a practical guide.

[b35] Lytle DA, Poff NL (2004). Adaptation to natural flow regimes. Trends Ecol. Evol.

[b36] Lytle DA, Bogan MT, Finn DS (2008). Evolution of aquatic insect behaviours across a gradient of disturbance predictability. Proc. Biol. Sci.

[b37] Martin L, Blossey B (2013). Intraspecific variation overrides origin effects in impacts of litter-derived secondary compounds on larval amphibians. Oecologia.

[b38] McCormick MI (2003). Consumption of coral propagules after mass spawning enhances larval quality of damselfish through maternal effects. Oecologia.

[b39] McCormick MI, Hoey AS (2004). Larval growth history determines juvenile growth and survival in a tropical marine fish. Oikos.

[b40] McMaster D, Bond NR (2008). A field and experimental study on the tolerances of fish to *Eucalyptus camaldulensis* leachate and low dissolved oxygen concentrations. Mar. Freshw. Res.

[b41] McNeil DG, Closs GP (2007). Behavioural responses of a south-east Australian floodplain fish community to gradual hypoxia. Freshw. Biol.

[b42] Metcalfe NB, Monaghan P (2001). Compensation for a bad start: grow now, pay later?. Trends Ecol. Evol.

[b43] Morrongiello JR, Bond NR, Crook DA, Wong BBM (2010). Nuptial coloration varies with ambient light environment in a freshwater fish. J. Evol. Biol.

[b44] Morrongiello JR, Bond NR, Crook DA, Wong BBM (2011). *Eucalyptus* leachate inhibits reproduction in a freshwater fish. Freshw. Biol.

[b45] Morrongiello JR, Bond NR, Crook DA, Wong BBM (2012). Spatial variation in egg size and egg number reflects trade-offs and bet-hedging in a freshwater fish. J. Anim. Ecol.

[b46] O'Connell M, Baldwin DS, Robertson AI, Rees G (2000). Release and bioavailability of dissolved organic matter from floodplain litter: influence of origin and oxygen levels. Freshw. Biol.

[b47] Poff NL, Allan JD (1995). Functional-organization of stream fish assemblages in relation to hydrological variability. Ecology.

[b48] Post DM (2003). Individual variation in the timing of ontogenetic niche shifts in largemouth bass. Ecology.

[b49] Quinn GP, Keough MJ (2002). Experimental design and data analysis for biologists.

[b50] R Development Core Team (2012). R: a language and environment for statistical computing.

[b51] Rasanen K, Laurila A, Merila J (2003). Geographic variation in acid stress tolerance of the moor frog, Rana arvalis. II. Adaptive maternal effects. Evolution.

[b52] Reid DJ, Quinn GP, Lake PS, Reich P (2008). Terrestrial detritus supports the food webs in lowland intermittent streams of south-eastern Australia: a stable isotope study. Freshw. Biol.

[b53] Rey D, David JP, Martins D, Pautou MP, Long A, Marigo G (2000). Role of vegetable tannins in habitat selection among mosquito communities from the Alpine hydrosystems. CR Acad. Sci. III-Vie.

[b54] Schlosser IJ (1990). Environmental variation, life-history attributes, and community structure in stream fishes: implications for environmental management and assessment. Environ. Manage.

[b55] Serrano L (1994). Sources, abundance and disappearance of polyphenolic compounds in temporary ponds of Donana National Park (South-western Spain). Aust. J. Mar. Freshw. Res.

[b56] Steinberg CEW, Kamara S, Prokhotskaya VY, Manusadzianas L, Karasyova TA, Timofeyev MA (2006). Dissolved humic substances - ecological driving forces from the individual to the ecosystem level?. Freshw. Biol.

[b57] Tableman M, Kim JS (2005). Survival analysis using S: analysis of time-to-event data.

[b58] Taborsky B (2006). The influence of juvenile and adult environments on life-history trajectories. Proc. Biol. Sci.

[b59] Temmink JHM, Field JA, Vanhaastrecht JC, Merkelbach RCM (1989). Acute and sub-acute toxicity of bark tannins in carp (*Cyprinus carpio* L.). Water Res.

[b60] Therneau TM, Granmbsch PM (2000). Modeling survival data: extending the Cox model.

[b61] Townsend SA, Edwards CA (2003). A fish kill event, hypoxia and other limnological impacts associated with early wet season flow into a lake on the Mary River floodplain, tropical northern Australia. Lakes Reserv. Res. Manage.

[b62] Watkins SC, Quinn GP, Gawne B (2010). Changes in organic-matter dynamics and physicochemistry, associated with riparian vegetation loss and river regulation in floodplain wetlands of the Murray River, Australia. Mar. Freshw. Res.

[b63] Watkins SC, Nielsen D, Quinn GP, Gawne B (2011). The influence of leaf litter on zooplankton in floodplain wetlands: changes resulting from river regulation. Freshw. Biol.

[b64] Weatherley AH, Gill HS (1987). The biology of fish growth.

[b65] Whitworth KL, Baldwin DS, Kerr JL (2012). Drought, floods and water quality: drivers of a severe hypoxic blackwater event in a major river system (the southern Murray–Darling Basin, Australia). J. Hydrol.

[b66] Wood CM, Matsuo AYO, Wilson RW, Gonzalez RJ, Patrick ML, Playle RC (2003). Protection by natural blackwater against disturbances in ion fluxes caused by low pH exposure in freshwater stingrays endemic to the Rio Negro. Physiol. Biochem. Zool.

[b67] Zuur AF, Ieno EN, Walker NJ, Saveliev AA, Smith GM (2009). Mixed effects models and extensions in ecology with R.

